# Application and Efficacy of Melatonin Elastic Liposomes in Photoaging Mice

**DOI:** 10.1155/2022/7135125

**Published:** 2022-03-08

**Authors:** Xueqin Hou, Xinyu Qiu, Yaping Wang, Shuangshuang Song, Yifan Cong, Jifu Hao

**Affiliations:** ^1^Institute of Pharmacology, Shandong First Medical University & Shandong Academy of Medical Sciences, Taian, Shandong 271016, China; ^2^College of Pharmacy, Shandong First Medical University & Shandong Academy of Medical Sciences, Taian, Shandong 271016, China

## Abstract

Transdermal drug delivery system is a preferable choice to overcome the low bioavailability of oral medication. Elastic liposomes have shown great effectiveness for percutaneous transport of melatonin (MLT). In this study, the elastic liposomes loaded with MLT were prepared using thin-film dispersion method and optimized through the central composite design (CCD) approach. The physicochemical properties and skin permeation against UV-induced skin photoaging efficacy of the developed MLT-ELs were assessed. The average size of the MLT-ELs was about 49 nm with a spherical shape and high encapsulation efficiency (73.91%) and drug loading (9.92%). The results of FTIR, DSC, and XRD revealed that the chemical structure of MLT was not changed after prepared elastic liposomes, and the drug was successfully encapsulated in the elastic liposome membrane material. In vitro skin permeation evaluation showed that the cumulative penetration of elastic liposomes was 1.5 times higher than that of conventional liposomes, highlighting that the elastic liposomes more easily penetrated into the body. The photoaging experiment results indicated that topical MLT-EL treatment ameliorated the skin elasticity, enhanced the skin hydration level, and preserved the integrity of dermal collagen and elastic fibers. It could be concluded that the elastic liposomes might serve as a promising platform for the transdermal delivery of melatonin.

## 1. Introduction

Melatonin (N-acetyl-5-methoxytryptamine, MLT), also known as pineal gland hormone, is a hormone synthetized in the pineal gland at nighttime through a sequence of well-known reactions from serotonin [1]. It plays a crucial role as a regulator of the sleep [2] and the immune response in humans [1]. MLT also has paracrine, autocrine, and antioxidant effects [3], and it can delay aging by act on the secretory system. Its protection action has also been reported in skin irradiation when included in topical formulations with ultraviolet filters [4]. However, its content is very low in the body, and it is important to supplement exogenous melatonin to maintain the normal physiological function of the body.

MLT is usually supplemented by oral administration, but most drugs are degraded through the liver due to the first-pass effect, and few can be used by the body [2]. In addition, it has a short half-life, low solubility, and instability, which limits its absorption in the body and affects the efficacy of ordinary melatonin preparations [2]. Therefore, it is proposed to change the way of drug delivery and exploit nanodelivery system, so as to increase the solubility of drug, prolong the half-life in vivo, and improve the bioavailability of drug.

Nanotechnology has a great influence on the development of new drug delivery systems or nanoplatforms. By using these nanoplatforms, different advantages can be obtained, such as selectively transforming the delivery mode of related drugs [5], changing the distribution in the body through different targeting strategies [6, 7], improving bioavailability [8], enhancing the efficiency of treatment [9], improving stability [10, 11], and increasing the absorption of certain drugs through different mucosal tissue [12, 13]. By the transdermal delivery routes, the local effects of nanoformulations can be enhanced to reach deeper layers of the skin; thus, the systemic bioavailability can be improved [14].

The transcutaneous route is attractive for drug delivery because it is suitable for cases of low bioavailability after oral administration, high first-pass effect in the liver, and inconvenient dosing regimens [15]. Compared with other drug delivery approaches, the percutaneous drug delivery system has many advantages, for example, it can effectively avoid the degradation of drugs in the liver, can maintain the drug therapeutic levels in the blood, has fewer side effects, and can achieve greater patient compliance with the treatment [15]. However, due to the unique “brick and mortar structure” barrier function of the stratum corneum (SC), many nanoformulations will stay in these structures so that it cannot penetrate deep into the skin, hindering most of therapeutic ingredients. Therefore, it is necessary to explore novel methods to weaken this protective effect of SC. Recently, a series of nanosystems has been researched, which were potential delivery vehicles for transdermal delivery, such as elastic liposomes, niosomes, and ethosomes [16–18].

Of all these available carrier systems, elastic liposomes (ELs) received extensive attention as transdermal delivery systems. Elastic liposomes are composed of lipids (usually phospholipids, cholesterol, etc.) and a biocompatible membrane softeners (such as sodium cholate, sodium deoxycholate, Tween, and Span) which have flexibility in the structure and it can encapsulate both hydrophilic and hydrophobic drugs in the phospholipid bilayer [18]. In addition, the membrane softeners are inserted in the middle of the phospholipid bilayer of elastic liposomes, which increased the distance between the phospholipid bilayer and disturbed the sequence of the phospholipid acyl chain, and its fluidity is increased so that it had more flexibility and deformation. In contrast to conventional liposomes (CLs) that are unable to penetrate deep into the skin and remain restricted to the SC, these elastic liposomes are able to penetrate into deeper skin layers [19]. Therefore, in order to improve the bioavailability of MLT and avoid the first-pass effect of the liver, it may be considered to change the dosage form and administration method, that is, we can prepare MLT into elastic liposomes for percutaneous delivery system.

In this study, we prepared elastic liposomes loaded with MLT by thin-film dispersion method. The formulation was optimized with the central composite design (CCD) response surface method, and their physicochemical properties were determined. The in vitro skin permeation of the developed elastic liposomes systems was evaluated by the modified Franz diffusion cell. Furthermore, we investigated whether topically applied with melatonin elastic liposomes could alleviate UV-induced skin photoaging through macroscopic, pinch test, skin hydration, histological, and MMP content evaluations.

## 2. Materials and Methods

### 2.1. Materials and Animals

Melatonin (MLT) was purchased from Shanghai Aladdin Biochemical Technology Co., Ltd. (Lot 118674, Shanghai, China). Phosphatidylcholine (PC) and cholesterol (Chol) were provided by Shanghai Aiweite Pharmaceutical Technology Co., Ltd. (Shanghai, China). Sodium deoxycholate (SDC) was obtained from Beijing Solable Technology Co., Ltd. (Beijing, China). Absolute ethanol was from Tianjin Kaitong Chemical Reagent Co., Ltd. (Tianjin, China). Chloroform was purchased from Fine Chemical Plant in Laiyang Economic and Technological Development Zone (Yantai, China). Methanol was provided by Tianjin Yongda Chemical Reagent Co., Ltd. (Tianjin, China). Sodium chloride injection was obtained from Henan Kelun Pharmaceutical Co., Ltd. (Henan, China). Paraformaldehyde was from Wuhan Sevier Biotechnology Co., Ltd. (Wuhan, China). Methanol of high-performance liquid chromatography (HPLC) grade was used, and all chemicals and other solvents used were of analytical grade.

Kunming female mice (35 ± 5 g) were obtained from the pharmacology laboratory of Shandong First Medical University (Shandong, China). All animals were handled according to the Principles of Laboratory Animal Care, and the protocols were approved by the Shandong First Medical University Animal Ethical Committee.

### 2.2. Preparation of Melatonin Elastic Liposomes (MLT-ELs)

The preparation of MLT-ELs was achieved via thin-film hydration method [20]. Briefly, the mixture with a fixed mass ratio of drug to PC and Chol (the different proportions of drug to PC and Chol are shown in Table 1) and SDC was codissolved in chloroform/ethanol (1 : 1 mixture). Subsequently, organic solvent was removed by evaporating at 40°C under vacuum to develop uniform thin film [21]. The resultant film was hydrated by rotary evaporating at 40°C for 1 h, which was then sonicated for 15 min using ultrasonic cell pulverizer to obtain MLT-ELs. The similar protocol was adopted to prepare melatonin conventional liposomes (MLT-CLs) except for incorporation of SDC and empty liposomes except for incorporation of model drug.

### 2.3. Optimization of MLT-ELs by CCD

After preliminary screening studies, two variables, that is, the amount of SDC (*A*) and the mass ratio of MLT to PC and Chol (*B*) in the elastic liposomes, were chosen to optimize formulation using dependent responses including drug loading (DL), entrapment efficiency (EE), and particle size (PS) as criteria [21]. The experimental schedule based on CCD is described in Table 1.

### 2.4. Determination of DL and EE

The content of MLT-ELs was determined by the HPLC method (Shimadzu LC-10AT, Tokyo, Japan). Briefly, the MLT-EL formulations were centrifuged at 8000 rpm for 20 min at 4°C temperature. The obtained free MLT solution was diluted to a certain concentration with methanol before determination. Then, the EE and DL of elastic liposomes were calculated using the following equations [22].

(1) $$\begin{array}{c} \text{EE}\left(\%\right)=\frac{\text{Weight of the feeding drug}-\text{Weight of the free drug}}{\text{Weight of the feeding drug}}\times 100\%,\\ \text{DL}\left(\%\right)=\frac{\text{Weight of the drug in elastic liposome }}{\text{Weight of the feeding lipid and drug}}\times 100\%. \end{array}$$

### 2.5. Physicochemical Characterization of MLT-ELs

#### 2.5.1. Average Particle Size (PS) and Polydispersity Index (PDI)

The PS and PDI of the prepared drug-free and drug-loaded elastic liposomes were measured by a dynamic light scattering apparatus (Zetasizer Nano S90, Malvern Instruments, UK) at room temperature (25°C ± 1°C) [23].

#### 2.5.2. Index Transmission Electron Microscopy (TEM)

The morphological profile of the prepared MLT-ELs and blank elastic liposomes (Blank-ELs) was observed by TEM (JEM-1200EX; JEOL, Tokyo, Japan). A drop of elastic liposomes was loaded onto a copper grid and then negatively stained with phosphotungstic acid solution (2%, *w*/*v*) for a while. After being air-dried at room temperature, the samples were examined under TEM [21, 23].

#### 2.5.3. Fourier-Transform Infrared Spectroscopic (FTIR) Measurements

The IR spectra of MLT, drug-free, and drug-loaded elastic liposomes were recorded on a FTIR spectrometer (IRAffinity-1S, Shimadzu, Japan) in the frequency range of 4000–400 cm^−1^. The chemical structure was analyzed based on the obtained infrared spectrum [23].

#### 2.5.4. Differential Scanning Calorimetry (DSC) of the Elastic Liposomes

The interaction between drugs and excipients was detected by DSC. All samples were scanned at the temperature range of 30-400°C/10°C/min under nitrogen atmosphere [24].

#### 2.5.5. X-Ray Powder Diffractometry (XRD) of the Elastic Liposomes

X-ray diffraction patterns were obtained with high-power XRD at intervals of 5°-45°/2*θ* [24].

### 2.6. In Vitro Skin Permeation Study

In vitro skin permeation of elastic liposome loaded with MLT was studied using Franz diffusion device with an effective diffusional area of 3.49 cm^2^ [23, 25]. In short, after the hair was removed in the abdomen of mice with an electric scissors, the full thickness of skin was cut from the hairless area. Then, the fatty tissue present in the dermis portion was removed. The obtained skin was mounted between the donor and receptor compartment of diffusion cell in such a way that stratum corneum side oriented to the donor side. The receptor chamber was filled with 6.5 ml phosphate buffer solution (PBS). The MLT-ELs (1.0 ml) was administrated in the donor compartment, whereas MLT-CLs (1.0 ml) were used as the control group. The medium of the receptor chamber was stirred using a magnetic rotor and maintained at 37°C ± 1°C. Three milliliters of the sample was withdrawn from receptor compartment at predetermined intervals (0.5, 1, 2, 3, 4, 6, 8, 10, and 24 h) and immediately replaced with equal volumes of fresh release medium. The obtained sample was measured at the wavelength of 223 nm by the ultraviolet spectrophotometer (UV-8000, METASH Shanghai, China). The cumulative permeated drug amount per unit area (*Q*) was calculated according to the following equation [23]:

(2) $$\begin{array}{c} Q=\frac{C_{n}\times V_{0}+\sum_{i=1}^{n-1}C_{i}\times V_{i}}{S}, \end{array}$$

where *Q* is the cumulative permeation amount per unit area, *S* is the effective diffusion area, *V*_0_ and *V*_*i*_ are the whole volume of the receptor compartment and the sample withdrawn volume at predetermined time point, respectively, and *C*_*i*_ and *C*_*n*_ represent the drug concentration in the release medium at time *i* and *n*, respectively.

Therefore, the linear regression curve of cumulative amount of drug that permeated through the skin (mg/cm^2^) against time (h) was plotted to describe drug permeation rate at steady state [23]. The drug flux at steady state (mg/(cm^2^·h)) was the slope of the regression line and calculated according to the equation

(3) $$\begin{array}{c} J_{\text{ss}}=\frac{dQ}{Sdt}. \end{array}$$

### 2.7. In Vivo Pharmacodynamic Studies

#### 2.7.1. Grouping of Animals

The thirty female Kunming mice were maintained under a natural light/dark cycle and fed with food and water ad libitum and adapted to the environment for one week before the experiment. At the beginning of the experiment, mice were randomly divided into five groups with six mice each. Dorsal skins of mice were shaved with an electric shaver for about 2.5 × 3 cm^2^ [26, 27]. Thereafter, the shaving was performed as required.

#### 2.7.2. Establishment of Photoaging Mouse Model

In order to establish the photoaging model, a self-made UV lamp irradiator was used. Mouse in the cages was irradiated under the UV lamp keeping the distance at 30 cm; it was irradiated for 2 h at the same time period each time and irradiated the next day for 10 weeks. After the UV irradiation, the shaved dorsal skins of mouse were applied topically with MLT-ELs or MLT-CLs every time for 10 weeks. And it was smeared 300 *μ*l each time on the skin of each mouse. Treatment schedule is shown in Table 2.

#### 2.7.3. Mouse Body Weight Changes

The body weight of mouse was weighed on Monday of each week during the entire experimental period, and the change curve of mouse body weight was drawn.

#### 2.7.4. Macroscopic Evaluation of Dorsal Skins

At the end of the experimental period, the UV-induced dorsal skin of each mouse which was under anesthesia was photographed; the grade of photodamage was determined referring to the grading scale shown in Table 3 [27]. The skin photoaging level ranged from 0 for normal skin to 6 for severely damaged skin.

#### 2.7.5. Evaluation of Recovery from Stretching (Pinch Test)

**Figure 1:**
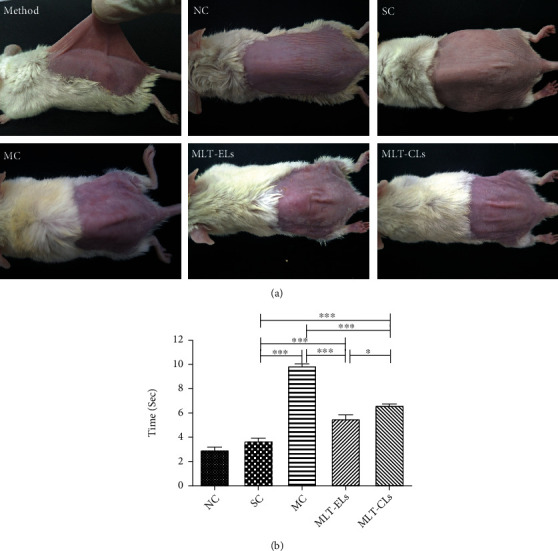
Evaluation of skin elasticity by pinch testing. (a) Photographs of pinch test. Method: the method of pinch test described by Tsukahara. (b) Recovery time was evaluated at the end of week 10. All data shown are mean ± SD of 6 mice; ^∗^*p* < 0.05 and ^∗∗∗^*p* < 0.0001. One-way ANOVA (*F* = 196.6, *p* < 0.0001) with *post hoc* Tukey's multiple comparison test was used for statistical analysis.

A pinch test was carried out on mouse referring to the method of Tsukahara [28]. Briefly, after the end of the experimental period, the midline of the dorsal skin of the anesthetized mouse was picked up with fingers as much as possible (to a degree that does not lift the animal into the air) and the pinch was subsequently released (see Figure 1(a)). And the skin recovery time was calculated immediately [26–29].

#### 2.7.6. Skin Hydration Measurement

At the end of the experimental period, the mice were sacrificed by cervical dislocation. The exposed skin on the back of the depilated mice was removed about 0.2 g. The fatty tissue present in the dermis portion was removed, and the wet weight of the skin was measured. Put the skin into an oven and dry to constant weight at 70°C, and then, weigh the dry weight of the skin. The skin hydration was calculated using the following equation:

(4) $$\begin{array}{c} \text{skin hydration}\left(\%\right)=\frac{M\left(\text{wet weight}-\text{dry weight}\right)}{M\left(\text{wet weight}\right)}\times 100\%. \end{array}$$

#### 2.7.7. Histopathology Studies

The skin samples were excised about 1.0 × 1.0 cm^2^ from the shaved dorsal skin of the mice immediately after they were sacrificed at the end of 10th week [27]. Then, samples were fixed with paraformaldehyde for at least 24 h, embedded in paraffin, and sliced with a microtome. The sections were stained with Hematoxylin-Eosin (H&E) staining [30] for routine histology study and stained with Victoria blue staining [31] to evaluate the elastic fibers in the skin of irradiated and nonirradiated mice. Finally, the structural changes of the stained specimens were observed under a light microscope (BX46, Olympus, Japan).

#### 2.7.8. Determination of MMP-1 and MMP-3 in the Skin

The dorsal skin tissue of mouse was homogenized (10,000 rpm, 20 s) in 9 volumes of PBS (4°C) to obtain the 10% homogenate and then centrifuged at 3000 rpm for 20 min at 4°C. According to the instructions, the total supernatant was utilized to estimate the secreted matrix metalloproteinase-1 (MMP-1) and matrix metalloproteinase-3 (MMP-3) by using enzyme-linked immunosorbent assay (ELISA) kits (Shanghai Enzyme-Linked Biotechnology Co., Ltd.) [32].

### 2.8. Statistical Analysis

All quantitative data were presented as the mean ± standard deviation (SD). Statistical analysis was performed using the GraphPad Prism 6 software. The changes in variable parameters between treated groups and the control group were analyzed by one-way analysis of variance (ANOVA) with *post hoc* Tukey's multiple comparison test. Differences were considered statistically significant if *p* < 0.05.

## 3. Results and Discussion

### 3.1. Optimization of MLT-ELs Based on CCD

The detailed experimental results obtained using CCD are listed in Table 4. The results are processed with regression analysis and described with quadratic polynomial equation models. And the three-dimensional response surface plots were constructed using Design-Expert software.

As shown in Table 4, the PS, EE, and DL of MLT-ELs ranged from 47.19 to 70.52 nm, 69.57% to 77.01%, and 5.42% to 10.13%, respectively. After statistical analysis, the results indicated that the regression equation model is significant, with *p* values of <0.0001. The regression equations of the fitted model constructed for PS, EE, and DL are presented below:

(5) $$\begin{array}{c} Y_{1}\left(\text{PS}\right)=75.569-4.587A-2.168B+5.000AB+0.291A^{2}+0.076B^{2},\\ Y_{2}\left(\text{EE}\%\right)=68.846-1.665A+0.469B+0.023AB-0.571A^{2}-0.011B^{2},\\ Y_{3}\left(\text{DL}\%\right)=3.201+1.485A-7.700B+0.025AB-0.032A^{2}-2.672B^{2}. \end{array}$$

**Figure 2:**
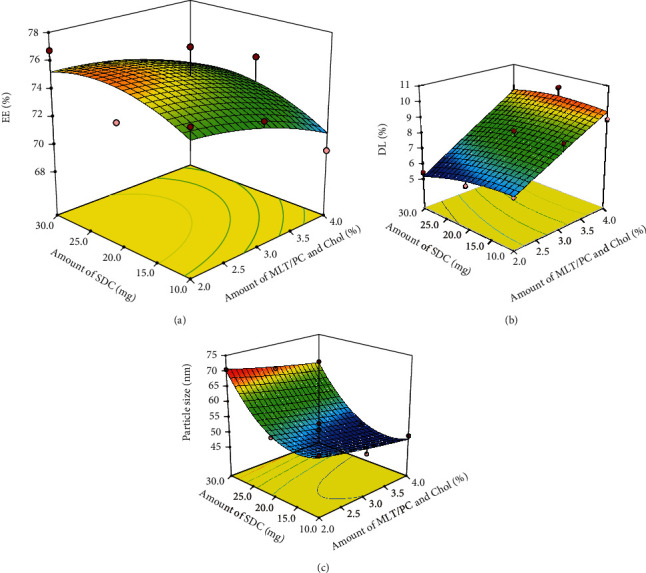
Response surface model showing the influence of the independent variables on the (a) EE, (b) DL, and (c) particle size. EE: encapsulation efficiency; DL: drug loading; MLT: melatonin; SDC: sodium deoxycholate; Chol: cholesterol; PC: phosphatidylcholine.

According to Figure 2, we observed that the PS changed slightly with the enhanced mass ratio of MLT to PC and Chol, while the EE decreased and the DL increased. This trend could be attributed to the fact that the particle size of elastic liposomes merely depended on the type of membrane softener. The size of the elastic liposomes remained a relatively stationary state once the elastic liposomes are formed. Therefore, the mass ratio of MLT to PC and Chol had a slight effect on the particle size. On the other hand, due to the saturation of the elastic liposome membrane-encapsulated drug, once the amount of drug fed exceeded the capability of the phospholipid, the amount of drug encapsulated in the elastic liposomes vesicles decreased and freed drug increased, resulting in the lowered EE and the augmented DL. Then, the DL did not show pronounced variance with the increase in the amount of SDC, while the EE first enhanced then decreased and the trend of PS was opposite to the EE. This might be related to the fact that the appropriate amount of membrane softeners was inserted into the phospholipid bilayer, the distance of the phospholipid molecules was incremented, the sequence of the phosphatidyl acyl chain was interfered, the fluidity of phospholipid bilayer was added, and the free drug reduced, resulting in the lowered PS and the increased EE. While the amount of SDC added exceeded, the structure of the phospholipid bilayer membrane structure was destroyed, resulting in the aggregation of elastic liposomes or leakage of drug, thus incremented PS and reduced EE.

After analyzing the effect of the independent variables on the dependent variables, a further optimization and validation strategy by the Design-Expert software optimized MLT-loaded elastic liposomes formulations, which depended on the prescriptive criteria of maximum EE and maximum DL and minimum PS. The composition of optimum formulation was determined as *A* = 18.91 mg (the amount of SDC) and *B* = 3.78% (the mass ratio of MLT to PC and Chol), which satisfied the target requirements. And the predicted values of PS, EE, and DL were 48.44 nm, 73.48%, and 9.16%, respectively. Therefore, in order to verify the predicted model, three new batches of elastic liposomes according to the optimal formulation were prepared. The detected optimal formulation had a PS of 49.47 ± 1.56 nm, an EE of 73.91 ± 0.13%, and a DL of 9.92 ± 0.03%, which was very consistent with the predicted value. The low deviation between these tested results and theoretical predictions indicates the dependability of the CCD used to predict the required elastic liposome formulation.

### 3.2. Characterization of MLT-ELs

#### 3.2.1. Particle Size and Morphology

**Figure 3:**
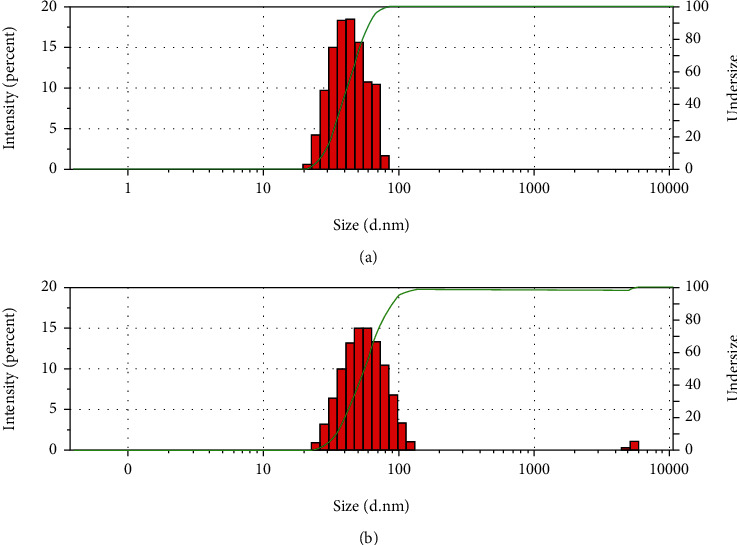
The particle size and size distribution of (a) MLT-ELs and (b) Blank-ELs.

The optimum elastic liposomes for loading MLT were successfully prepared and then subjected to physicochemical characterization. As shown in Figure 3, the mean particle size of the drug-loaded and drug-free elastic liposomes was about 49 nm and 69 nm and the PDI were 0.203 and 0.260, respectively. Relational researches indicated that only sizes of 50 to 500 nm particles were possible to penetrate into the skin [33], and the smaller sizes of particles can make drug across the barrier of the stratum corneum easily. The results displayed the smaller particle size and narrower size distribution both of the elastic liposomes loading with or without the drug, manifesting that elastic liposome formulations were suitable for transdermal delivery.

#### 3.2.2. TEM Analysis

**Figure 4:**
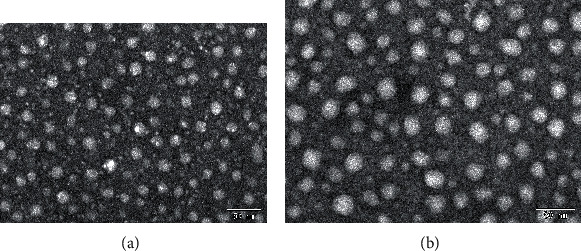
TEM images of the (a) MLT-ELs and (b) Blank-ELs.

The morphologies of the elastic liposomes loading with or without the MLT were observed by TEM (Figure 4). The results displayed that the prepared elastic liposomes were homogeneous spherical globules without aggregation. The particle size of elastic liposomes was smaller by TEM than the dynamic light scattering apparatus. One of the possible reasons was that the hydrodynamic diameter of liposomes was reflected via dynamic light scattering apparatus and the diameter of liposomes after drying was observed with TEM, so that had a certain degree difference of particle size measured by these two manners.

#### 3.2.3. IR Study

**Figure 5:**
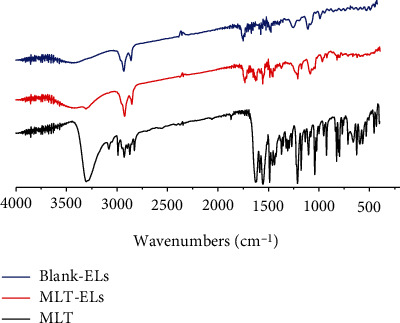
FTIR spectrum of Blank-ELs, MLT-ELs, and MLT.

In order to investigate the possible drug-excipient interaction, an FTIR analysis was performed. As shown in Figure 5, FTIR spectra included MLT powder, MLT-laden elastic liposomes, and blank elastic liposomes. The IR spectrum of MLT-laden elastic liposomes was greatly similar to the blank elastic liposomes, whereas it was distinctly different from that of the MLT power. The FTIR spectrum of MLT stretching peaks was determined at 3333.59 cm^−1^, 1625 cm^−1^, and 1210 cm^−1^ associated with the functional N-H stretch, C=O, and C-N, respectively [34, 35]. However, these typical peaks disappeared in the spectrum of the elastic liposomes. The resemblance of IR spectrum between drug-laden elastic liposomes and drug-free elastic liposomes indicated that the drug and excipient has favorable compatibility during the preparation of elastic liposomes.

#### 3.2.4. DSC Analysis

**Figure 6:**
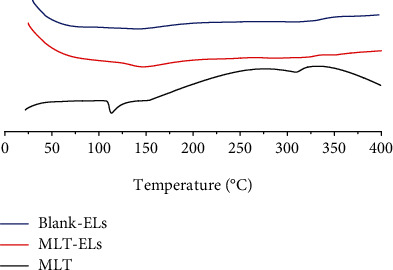
DSC result of Blank-ELs, MLT-ELs, and MLT.

DSC patterns of MLT powder, MLT-loaded elastic liposomes, and blank elastic liposomes are shown in Figure 6. The results displayed that endothermic peak of MLT was observed at about 116°C, which is due to the melting of MLT, revealing its crystalline structure. Previous studies indicated a remarkable endothermic peak of MLT at 119°C [34]; this result was basically consistent with the literature results. And there was disappearance of melting endotherm peak of MLT in elastic liposomes; this may be due to the existence of certain interactions in the elastic liposomes, which led to the disappearance of the melting peak and the change of the crystal structure of MLT. This result demonstrated that a fully amorphous and uniform distribution of MLT was presented in elastic liposomes.

#### 3.2.5. XRD Study

**Figure 7:**
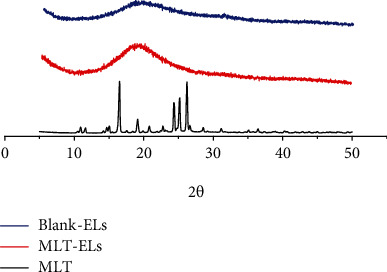
XRD patterns of Blank-ELs, MLT-ELs, and MLT.

Figure 7 presents the XRD results for MLT powder, MLT-laden elastic liposomes, and blank elastic liposomes. MLT exhibited sharp diffraction peaks at 2*θ* = 16.5°, 19.1°, 24.3°, 25.2°, and 26.2°, suggesting that it has a crystalline structure [36, 37]. The XRD pattern of MLT-laden elastic liposomes and blank elastic liposomes showed that it did not exhibit the sharp diffraction peaks of MLT, but presented a broad peak at the range of 15°-30°, confirming its amorphous characteristic. It indicated that a great amount of amorphous material was formed during the preparation of elastic liposomes. In addition, this result was similar to that observed with DSC. The disappearance of crystalline peaks provides evidence for the formation of elastic liposomes [36].

### 3.3. In Vitro Skin Permeation Study

**Figure 8:**
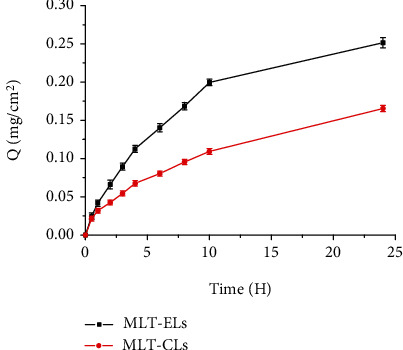
In vitro cumulative release profiles of MLT through mouse skins.

The in vitro cumulative permeation characteristics of MLT released from the elastic liposomes and conventional liposomes were assessed through isolated dermis of mouse. As shown in Figure 8, the cumulative permeation amount per unit area of permeated drug in the receptor compartments increased with time. MLT released from two different liposomes appeared the similar models of skin permeation curves, while the cumulative penetration of elastic liposomes was higher than that of conventional liposomes. The steady-state fluxes (*J*_ss_) and other parameters with different liposome formulations are summarized in Table 5. The elastic liposomes displayed higher cumulative permeation and transdermal flux of 0.2514 ± 0.0448 mg/cm^2^ and 0.0176 ± 0.0042 mg/cm^2^/h, respectively, whereas the conventional liposomes were found to have cumulative permeation and flux of 0.1655 ± 0.0356 mg/cm^2^ and 0.0087 ± 0.0035 mg/cm^2^/h, respectively. The cumulative permeation and transdermal flux of elastic liposomes were about 1.5-fold and 2-fold higher than those of conventional liposomes, respectively. Furthermore, the lag time of elastic liposomes (1.5341 h) was significantly shorter than that of conventional liposomes (2.6322 h). These results explained that elastic liposome formulas had more favorable skin permeation capacity than conventional liposomes.

These results may be explained by the fact that the SDC as membrane softener used in the elastic liposomes altered the sequence of phosphatidyl chains and increased the fluidity of phospholipid bilayer so that the vesicle membrane had a higher elasticity [38]. On the other hand, the phospholipid of the liposome membrane material had good biocompatibility with the phospholipid bilayer of the stratum corneum and disturbed the protective barrier of the stratum corneum [38]. And the elastic liposome entered the subcutaneous tissue through the stratum corneum; it could still maintain its original shape to ensure that more drugs could enter the body to exert the therapeutic effect.

### 3.4. In Vivo Pharmacodynamic Studies

#### 3.4.1. The Body Weight Change Analysis

**Figure 9:**
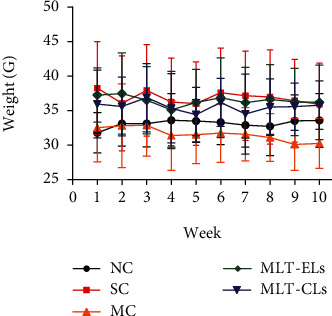
Change in body weight of UV-irradiated mice with different treatments.

The body weight curve of mice in Figure 9 showed that there was no manifestation of the body weight gain of each group during the entire experimental period. This might be attributed to individual differences between the mice or artificial measurement bias. Due to the changes of the surrounding environment such as ultraviolet radiation, which may affect the physical development of the mouse, we could determine whether the ultraviolet radiation would affect the physical functions of the mice by recording the changes of body weight during the experiment. The results indicated that ultraviolet radiation would not affect the normal vital signs of mouse.

#### 3.4.2. MLT-ELs Reduced UV-Induced Macroscopic Skin Lesions

**Figure 10:**
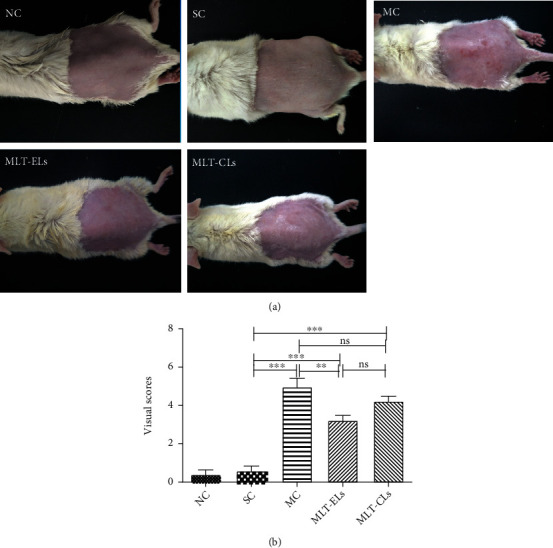
MLT-ELs and MLT-CLs prevent UV-induced macroscopic skin lesions in mouse. (a) Physical appearances of UV-irradiated mouse with various treatments at the last week. (b) Results of the visual score of different experimental groups at the last week. All data shown are mean ± SD of 6 mice; ^∗∗^*p* < 0.001, ^∗∗∗^*p* < 0.0001, ns: no significance. One-way ANOVA (*F* = 48.75, *p* < 0.0001) with *post hoc* Tukey's multiple comparison test was used for statistical analysis.

Macroscopic effects of UV irradiation on mouse skin are displayed in Figure 10. As shown in Figure 10(a), at the end of the experiment, the mice in the NC group and the SC group showed that there were no erythemas and leathery appearance, while the mouse in the SC group displayed slightly rough appearance than that in the NC group. This tiny change can be seen with the naked eye, but could not be observed in both the macroscopic and histological photographs. We think this might be the result of shaving and an effect perhaps masked by the lesions caused by UV radiation in other groups [26]. This indicated that shaving did not cause macroscopic skin damage. Skin erythemas and leathery appearance were observed in MC group mouse, whereas application with MLT-ELs and MLT-CLs had a tendency to inhibit the UV-induced extensive erythemas after treatment. And the mouse in the MLT-EL group revealed more smooth appearance than that in the MLT-CL group.

Statistically, Figure 10(b) manifests that the visual scores of the NC and SC groups had no significant difference (*p* > 0.05), but both were distinctly lower than the other three groups. Although the scores of the MLT-EL group and the MLT-CL group showed no statistical difference (*p* > 0.05), these two groups were markedly reduced compared with the MC group. These results demonstrated that ultraviolet irradiation for 10 weeks caused mouse skin lesions, but use of melatonin liposome formulations, especially MLT-ELs, could prevent these macroscopic damages.

#### 3.4.3. MLT-ELs Returned the Skin Elasticity in the Pinch Test

At the end of treatment for 10 weeks, the time that the mouse skin returned from stretch was recorded by the pinch test. Figure 1(a) shows photographs of dorsal skins of various mouse groups after being stretched for 1 sec. As described in Figure 1(b), the recovery times of the mice needed in the NC group and the SC group were not significantly different (*p* > 0.05), but both were obviously shorter than the other three groups. Moreover, the recovery time of the MLT-EL group and the MLT-CL group displayed had shortened compared with that of the MC group. According to relevant literature, UV-induced photoaging is mainly manifested as decreased skin elasticity [39, 40]. Our results showed that the recovery time was significantly prolonged when the mouse skin was exposed to UV for 10 weeks, indicating that skin elasticity was markedly reduced. However, MLT-laden liposome treatment obviously shortened the recovery time. These data cleared that treatment with MLT-ELs could improve the skin elasticity by ultraviolet radiation, and its effects were better than MLT-CLs.

#### 3.4.4. MLT-ELs Increased the Skin Hydration

**Figure 11:**
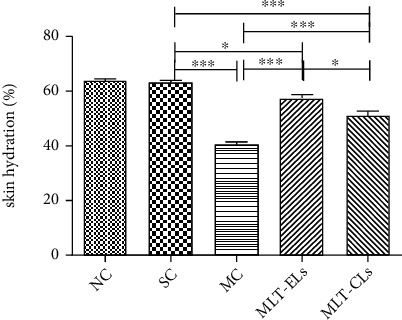
Evaluation of skin hydration of UV irradiation on mice. All data shown are mean ± SD of 6 mice; ^∗^*p* < 0.05 and ^∗∗∗^*p* < 0.0001. One-way ANOVA (*F* = 68.97, *p* < 0.0001) with *post hoc* Tukey's multiple comparison test was used for statistical analysis.

The skin hydration of UV irradiation on mouse in Figure 11 demonstrated that there were no significant differences (*p* > 0.05) in the NC group and the SC group, but the other three groups were statistically different (*p* < 0.05) from the SC group. And the skin hydration in the NC group and the SC group was obviously higher than that in the other three groups. In addition, hydration of the skin in the MLT-EL and MLT-CL groups showed a remarkable increase (*p* < 0.05) when compared with that in the MC group. It was well known that long-term exposure to UV could cause skin dryness, so the antiphotoaging effect of melatonin liposome formulations could be determined by measuring the skin hydration. And related research has shown that the skin hydration of mice irradiated with UV would be reduced [41], which was basically consistent with our results. These results revealed that treatment with melatonin liposome preparation was able to elevate the skin hydration level, and MLT-ELs had better effect than MLT-CLs.

#### 3.4.5. MLT-ELs Mitigated the Structural Damage of the Skin

**Figure 12:**
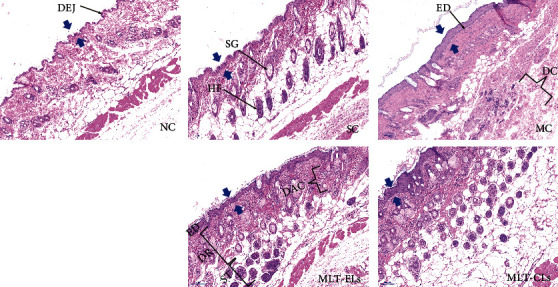
Photographs of mouse skin tissue observed by H&E staining: NC group (×100); SC group (×100); MC group (×100); MLT-ELs (×100); MLT-CLs (×100). DEJ: dermal–epidermal junction; ED: epidermis; DR: dermis; ST: subcutaneous tissue; HF: hair follicle; SG: sebaceous glands; DAC: densely arranged collagen fibers; DC: degraded collagen fibers which were messily arranged.

**Figure 13:**
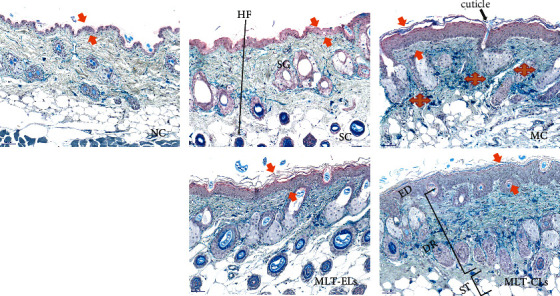
Photographs of mouse skin tissue observed by Victoria blue staining: NC group (×200); SC group (×200); MC group (×200); MLT-ELs (×200); MLT-CLs (×200). ED: epidermis; DR: dermis; ST: subcutaneous tissue; HF: hair follicle; SG: sebaceous glands.

The histopathology alterations of mouse skin induced by UV are displayed in Figures 12 and 13. As demonstrated in Figure 12, the skin features of the mice in the NC group and the SC group were similar, which showed a normal and complete structure, and the subcutaneous hair follicles and sebaceous glands were plump and complete. To be specific, the epidermal tissue structure was complete and the boundary of each cell layer was clear in the two groups. The epidermis was relatively thin, and the thickness of which was uniform. The dermal–epidermal junction (DEJ) was a wavy thin line of cells between the dermis and epidermis that could be clearly observed along with the epidermal projection and dermal papilla (DP). In addition, the dermis displayed an ordered arrangement of undulating collagen fibers, and its thickness and distribution were uniform. Moreover, the cell composition and quantity of the dermis were moderate.

In the MC group, it showed irregular epidermal hyperplasia, incomplete structure, and unclear stratification. Furthermore, the DEJ was a straight line between the epidermis and dermis and disappearance of epidermal projection and dermal papillae. In the dermis, it shrinks and becomes thinner, the cell arrangement was disordered and even disintegrates, the collagen fiber bundles showed disorganized and loose, and some of which even showed destruction and accompanied by inflammatory cell infiltration.

The skin condition of the MLT-CL group and the MLT-EL group recovered to a certain extent compared with that of the MC group. The skin of the mouse treated with MLT-CLs showed thickened epidermis and dermis. Hair follicles and sebaceous glands with relatively complete structure could be observed. The dermis displayed which tended to be an orderly arrangement of collagen fibers, although some of these were scattered. Inflammatory infiltrates still exist in the dermis. The skin structure of the MLT-EL group tended to be complete, and it showed mild hyperplasia of the epidermis. The structure of the DEJ returned to normal. Meanwhile, the number of collagen fiber bundles in the dermis was increased and thickened, and they displayed an ordered arrangement. These results indicated that the melatonin liposome preparation, especially MLT-ELs, had good biocompatibility and it also mitigated the structural damage of the skin.

It also could be seen from Figure 13 that the elastic fiber structure in the skin of the NC group and the SC group was intact (elastic fiber network with clear structure could be seen) and arranged orderly. After UV irradiation group (MC group), the elastic fibers of the mouse skin thickened, broke, and twisted, and some elastic fibers had aggregated, tangled, and disordered arrangement. The above results indicated that chronic application of ultraviolet radiation could damage the elastic fibers of the skin of the test mice and undermine their network physiological structure, indicating that this experiment successfully established the mouse photoaging model. MLT-EL and MLT-CL group elastic fibers in the skin had different degrees of recovery and were arranged orderly. Occasionally, it could see the phenomenon of aggregation, breakage, and tangle. After comparison, it was found that the effect of MLT-ELs against UV damage to skin elastic fibers was closer to its normal morphology and physiological structure.

Related research suggested that skin aging could cause changes in the arrangement and structure of collagen fibers [26]. In this study, it was found that the arrangement of collagen fibers was seriously disturbed in mouse skin after long-term UV exposure. MLT-EL treatment could maintain the integrity of collagen fiber structure in irradiated mouse skin. These results demonstrated that MLT-ELs help prevent skin aging, which might be mainly caused by disordered arrangement and structural changes of collagen fibers in the skin.

In addition, elastic fibers were the key to maintain the elasticity and tension of the skin. Once the elastic fibers were damaged or broken, they could directly damage the network structure of the dermis layer, leading to the significant reduction of skin elasticity and the formation of sagging skin [26, 42]. In this experiment, Victoria blue staining was used to further compare the elastic fibers in mouse skin of normal and irradiated with UV, so as to investigate whether MLT-ELs have a protective effect on elastic fibers of the mouse skin. It was discovered that normal structure of skin elastic fibers was replaced by large quantities of thickened, tangled, and broken fibers when mouse skin was exposed under UV for 10 weeks. However, MLT-EL treatment reversed the disrupted elastic fibers caused by UV. These findings were in accordance with the results in pinch test and manifested that MLT-ELs had the potential to keep the integrity of elastic fibers in the dermis and could further alleviate chronic UV-induced skin sagging and reduction of elasticity.

#### 3.4.6. MLT-ELs Inhibited the Secretion of MMPs in Mouse Skin

**Figure 14:**
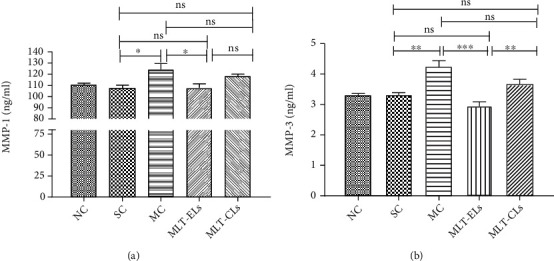
Determination of (a) MMP-1 and (b) MMP-3 contents of UV irradiation on mouse skin. All data shown are mean ± SD of 6 mice; ^∗^*p* < 0.05, ^∗∗^*p* < 0.001, ^∗∗∗^*p* < 0.0001, ns: no significance. One-way ANOVA ((a) *F* = 4.717, *p* = 0.0067; (b) *F* = 11.7, *p* < 0.0001) with *post hoc* Tukey's multiple comparison test was used for statistical analysis.

The expression levels of MMPs in the skin tissues of mice irradiated with UV are shown in Figure 14. The levels of MMP-1 and MMP-3 in the skin tissues of the NC group and the SC group were close to each other, and there were no significant difference (*p* > 0.05). Compared with the SC group, the levels of MMP-1 and MMP-3 in the MC group were significantly increased (*p* < 0.05). The contents of MMP-1 and MMP-3 in the skin tissues of mice in the MLT-EL group and the MLT-CL group were lower than those in the MC group. The results revealed that the melatonin liposome preparation can significantly inhibit the increase of MMP-1 and MMP-3 secretion in the skin of mice caused by UV exposure, and the effect of MLT-ELs was superior to MLT-CLs.

Matrix metalloproteinases (MMPs), a group of zinc-dependent extracellular proteinases, especially MMP-1 and MMP-3, break down the extracellular matrix (ECM), which is an essential structural framework for skin cells and mainly includes collagen and elastic fibers [32]. In short, MMP-1 can degrade collagen fibers of types I and III; after these two types of collagen fibers are cut by MMP-1, MMP-3 can continue to degrade this broken collagen fibers and it can also activate other MMPs, thus degrading various ECM such as elastic fibers in the dermis. Studies have confirmed that the levels of MMP-1 and MMP-3 were increased in UV-irradiated mouse skin [32, 43], so it plays an important role in skin photoaging. In the present study, our results were basically consistent with the results reported in the literature [32, 43]. In addition, the reliability of the results was further confirmed by the pinch test and histopathology experiment. Therefore, there is reason to believe that MLT-ELs is an effective MMP inhibitor, which can significantly inhibit the expression of MMP-1 and MMP-3, thereby reducing the degradation of collagen and elastic fibers, and then protect the skin from damage caused by ultraviolet radiation.

## 4. Conclusion

In this study, a functional MLT-loaded elastic liposome platform was developed through thin-film dispersion approach and applied for delivery of MTL through the skin to achieve antiaging effect caused by UV radiation. As a result of the formulation optimization, the elastic liposomes exhibited spherical shape, small size, and high DL and high EE. In vitro skin permeation studies indicated that elastic liposomes can enhance the transdermal delivery of MLT. The results of mouse skin photoaging model demonstrated that topical MLT-EL treatment improved the skin elasticity, increased the skin hydration level, and maintained the integrity of dermal collagen and elastic fibers. In summary, these results clearly prove the protective effects of topical administration of MLT-ELs on photoaging mouse skin caused by ultraviolet radiation and indicate it may help prevent photoaging.

## Figures and Tables

**Table 1:** Variables and their levels in the CCD.

Independent variables	Levels		
	-1	0	1
*A* = amount of SDC (mg)	10	20	30
*B* = mass ratio of MLT to PC and Chol (%)	2	3	4
Dependent variables	Constraints		
*Y*_1_ = particle size (nm)	Minimize		
*Y*_2_ = entrapment efficiency (%)	Maximize		
*Y*_3_ = drug loading (%)	Maximize		

Abbreviations: CCD: central composite design; SDC: sodium deoxycholate; Chol: cholesterol; PC: phosphatidylcholine.

**Table 2:** Treatment schedule of the study.

Group	Shave	UV radiation	MLT-ELs	MLT-CLs
			300 *μ*l/mouse	
Naïve control (NC)	-	-	-	-
Sham control (SC)	+	-	-	-
Model control (MC)	+	+	-	-
Melatonin elastic liposomes (MLT-ELs)	+	+	+	-
Melatonin conventional liposomes (MLT-CLs)	+	+	-	+

-: without treatment; +: with treatment.

**Table 3:** Grading scale for evaluation of photoaging.

Grade	Evaluation criteria
0	Smoothness without any wrinkles; fine striations running the length of the body
1	Fine striations
2	A few shallow wrinkles; disappearance of all fine striations
3	Shallow wrinkles across the dorsal skin
4	Deep and coarse wrinkles with laxity
5	Increased deep wrinkles
6	Surface accompanied with severe wrinkles; development of lesions

**Table 4:** Experimental runs and results of MLT-ELs based on CCD.

No.	SDC (mg)	MLT/(PC and Chol)	PS (nm)	EE (%)	DL (%)
1	10	2	54.05	74.75	6.22
2	30	2	70.52	76.75	5.42
3	10	4	48.86	69.57	8.88
4	20	3	50.92	77.01	8.14
5	20	2	53.82	73.32	5.70
6	30	4	65.35	72.49	9.08
7	20	3	52.98	72.14	7.08
8	20	3	50.92	77.01	8.14
9	20	3	52.98	72.14	7.08
10	20	4	47.19	74.95	10.13
11	20	3	50.92	77.01	8.14
12	10	3	48.62	73.41	8.36
13	30	3	66.91	73.75	7.00

Abbreviations: CCD: central composite design; MLT: melatonin; SDC: sodium deoxycholate; Chol: cholesterol; PC: phosphatidylcholine.

**Table 5:** Percutaneous permeation parameters of MLT-ELs and MLT-CLs.

Formulations	Cumulative permeation	Kinetic equations	Lag time (h)	Flux *J*_ss_ (mg·cm^−2^·h^−1^)	Permeability coefficient
MLT-ELs	0.2514 ± 0.0448	*Q* = 0.0176 *t*–0.0270	1.5341	0.0176 ± 0.0042	0.0093 ± 0.0022
MLT-CLs	0.1655 ± 0.0356	*Q* = 0.0087 *t*–0.0229	2.6322	0.0087 ± 0.0035	0.0046 ± 0.0019

## Data Availability

The data are available from the corresponding author upon request.
